# A Haplotype-Based GWAS Identified Trait-Improving QTL Alleles Controlling Agronomic Traits under Contrasting Nitrogen Fertilization Treatments in the MAGIC Wheat Population WM-800

**DOI:** 10.3390/plants11243508

**Published:** 2022-12-14

**Authors:** Antonia Lisker, Andreas Maurer, Thomas Schmutzer, Ebrahim Kazman, Hilmar Cöster, Josef Holzapfel, Erhard Ebmeyer, Ahmad M. Alqudah, Wiebke Sannemann, Klaus Pillen

**Affiliations:** 1 Institute of Agricultural and Nutritional Sciences, Martin-Luther-University Halle-Wittenberg, Betty-Heimann-Str. 3, 06120 Halle, Germany; 2 Syngenta Seeds GmbH, Kroppenstedter Str. 4, 39387 Oschersleben, Germany; 3 RAGT 2n, Steinesche 5A, 38855 Wernigerode, Germany; 4 Secobra Saatzucht GmbH, Feldkirchen 3, 85368 Moosburg an der Isar, Germany; 5 KWS Lochow GMBH, Ferdinand-Lochow-Str. 5, 29303 Bergen, Germany; 6 Biological Science Program, Department of Biological and Environmental Sciences, College of Art and Science, Qatar University, Doha P.O. Box 2713, Qatar

**Keywords:** multi-parent advanced generation intercross (MAGIC), single nucleotide polymorphism (SNP), haploblock, haplotype, genome-wide association study (GWAS), quantitative trait locus (QTL), grain yield, nitrogen fertilization, wheat breeding

## Abstract

The multi-parent-advanced-generation-intercross (MAGIC) population WM-800 was developed by intercrossing eight modern winter wheat cultivars to enhance the genetic diversity present in breeding populations. We cultivated WM-800 during two seasons in seven environments under two contrasting nitrogen fertilization treatments. WM-800 lines exhibited highly significant differences between treatments, as well as high heritabilities among the seven agronomic traits studied. The highest-yielding WM-line achieved an average yield increase of 4.40 dt/ha (5.2%) compared to the best founder cultivar Tobak. The subsequent genome-wide-association-study (GWAS), which was based on haplotypes, located QTL for seven agronomic traits including grain yield. In total, 40, 51, and 46 QTL were detected under low, high, and across nitrogen treatments, respectively. For example, the effect of QYLD_3A could be associated with the haplotype allele of cultivar Julius increasing yield by an average of 4.47 dt/ha (5.2%). A novel QTL on chromosome 2B exhibited pleiotropic effects, acting simultaneously on three-grain yield components (ears-per-square-meter, grains-per-ear, and thousand-grain-weight) and plant-height. These effects may be explained by a member of the nitrate-transporter-1 (NRT1)/peptide-family, *TaNPF5.34*, located 1.05 Mb apart. The WM-800 lines and favorable QTL haplotypes, associated with yield improvements, are currently implemented in wheat breeding programs to develop advanced nitrogen-use efficient wheat cultivars.

## 1. Introduction

Wheat (*Triticum aestivum* L.) is one of the most commonly grown crops worldwide following after maize and rice [1], thus playing a major role in the sustainable increase of human food supply [2]. Boosting gain yield in wheat is imperative to satisfy future wheat demands [3] that are estimated to increase from 760 to 900 million tons by 2050 [4]. A solution must be found for this ambivalent situation, which is at the heart of research today. One way of achieving more sustainable agriculture is the reduction of fertilizer, along with a decrease in soil acidity, which is only one example of many serious threats to food security worldwide [5]. Previous observations indicate that the long-term use of nitrogen (N) fertilization causes soil acidity, which consequently leads to a decrease in wheat yields [6]. The inefficient use of N fertilizer is a global problem that increases production costs, environmental pollution, and the emission of greenhouse gases [7]. Therefore, new adapted crops need to be developed with a higher nitrogen use efficiency and strong yield stability.

In general, nitrogen use efficiency (NUE) refers to a crop plant’s ability to use available N in soils to produce grain yield per unit area. It consists of N uptake efficiency and N utilization efficiency of the absorbed N [8]. Therefore, it is crucial to understand the molecular basis of plant responses to N to enable further yield improvements and adaptations of plants under changing N conditions. There is a substantial body of research on the improvement of NUE in wheat [9,10,11,12,13,14,15] and many studies concentrated on N uptake and remobilization mechanisms in plants [16,17,18]. Developing high NUE in wheat cultivars can be achieved through conventional breeding and/or genetic improvement by manipulating NUE genes. Mapping of quantitative trait loci (QTL) is an effective approach to understanding the genetic basis of NUE and identifying those genes underlying NUE. For wheat, genetic studies using recombinant inbred line (RIL) populations assisted in detecting NUE QTL under field conditions [15,19]. Furthermore, previous studies on wheat reported N-dependent effects and diverging transcription levels of multiple nitrate transporters [20,21,22]. In addition to QTL, several genes controlling NUE related-traits have been identified in wheat including *TaAS1-3A*, *TaAS1-3D*, *TaASN2-1A*, *TaASN2-1B*, *TaANR1-6A*, *TaANR1-6B*, *TaNRT2.4-6A*, *TaNRT2.6-6A*, *TaNRT2.6-6B* and *TaPHT1.5-5B* [7,23]. Two transporter gene families are known in higher plants (*NRT1* and *NRT2*) to be involved in the low- and high-affinity nitrate transport systems (LATS and HATS). Moreover, two amino acid synthetase gene families (asparagine synthetase and glutamine synthetase) are also involved in NUE [23]. These findings support the assumption that wheat may respond to contrasting N applications by inducing or repressing various sets of genes.

A genome-wide association study (GWAS) based on high-density molecular markers has been intensively used in crop plants as a powerful tool for the genetic dissection of complex traits [24]. GWAS relies on the association between the molecular marker and the phenotype of a target trait. For NUE in wheat, few GWAS scans have been conducted to detect markers associated with NUE-related traits and to explore the genetic basis using diverse genetic collections under contrasting N conditions [25,26]. However, QTL identified by GWAS do not represent true causal molecular variants due to limitations in biallelic single-nucleotide polymorphism (SNP). Therefore, haplotype (HT)-based GWAS is an effective approach to increase the allelic variation and resolution of associated genomic regions based on haplotype blocks made of two or more SNPs in high LD [27]. The haplotype approach is instrumental to reduce the complexity in multi-parental populations and assigning allelic effects to a particular founder.

To find high genetic variation, multi-parental populations are promising to increase genetic diversity, which can be used for high-resolution trait mapping [28]. In comparison to traditional bi-parental populations, multi-parental populations combine a broad allele diversity [29] and a high number of recombination events. The design of multi-parent advanced generation intercross (MAGIC) populations is a valuable genetic resource and is recommended for both crop breeding and quantitative genetic studies [28]. So far, a number of MAGIC populations have been developed for crop species and model plants and used in quantitative genetics studies to support breeding and functional gene analysis [30,31,32,33,34,35,36,37,38,39,40,41,42,43].

The eight-way MAGIC-WHEAT population WM-800, developed through intercrossing eight elite winter wheat founders, revealed a valuable tool for the genetic dissection of important agronomic traits [44]. With a population size of 800 lines, it is among the biggest MAGIC populations developed so far supporting its statistical power to identify QTL. In this study, we investigated the genetic architecture of N-dependent QTL effects on yield-related traits using the MAGIC-WHEAT population WM-800. For this, field trials with WM-800 and controls were conducted at four locations in Germany during two seasons under two contrasting N fertilization treatments. Up to our knowledge, we present the first genetic study of agronomic traits under low and high nitrogen treatments in wheat using a MAGIC population and applying a haplotype-based GWAS.

## 2. Results and Discussion

### 2.1. Phenotypic Variation in WM-800 Cultivated under Two Contrasting N Fertilization Treatments

In the current study, we examined the effect of contrasting N fertilization treatments on developmental and yield-related traits in the MAGIC-WHEAT population WM-800. The study was carried out at four locations and during two consecutive growing seasons. To our knowledge, this is the first time that the studied traits were evaluated under contrasting N treatments in a MAGIC wheat population. We could observe strong phenotypic variation among the WM-800 lines as an effect of N treatment in addition to environmental influence (Table 1, Tables S4 and S5).

**Figure 1:**
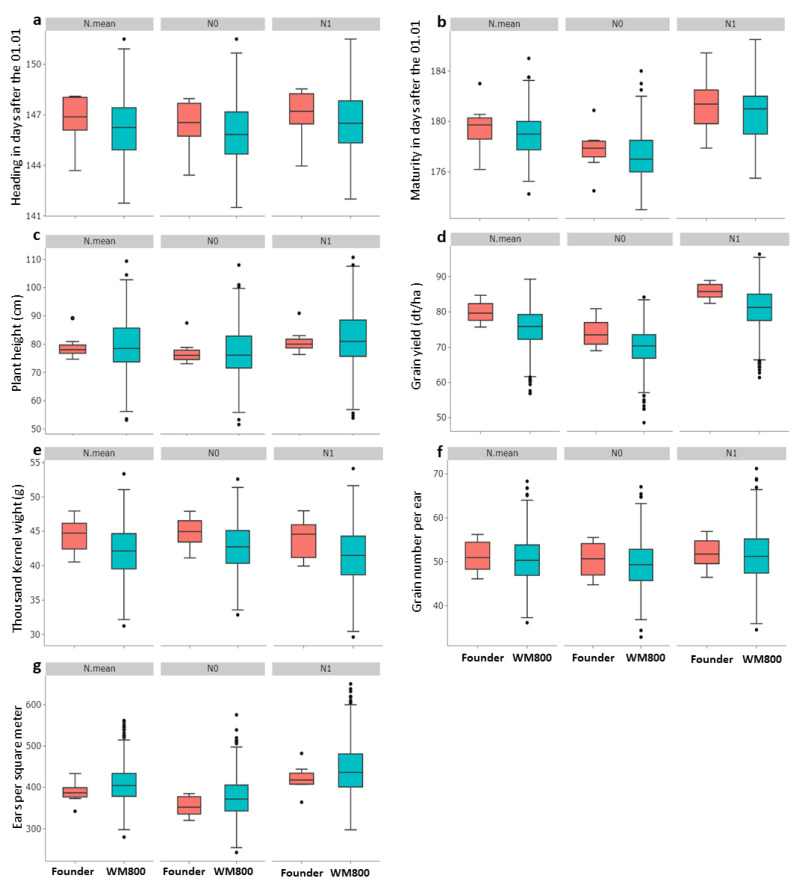
Box-Whisker-Plots indicating variations among WM-800 lines and founders across (N.mean) and within N treatments (N0 and N1) for seven traits studied (subplots (**a**–**g**)). For all traits, the ANOVA indicated highly significant differences between N treatments at *p* < 0.001 or *p* < 0.01 for both population WM-800 and the founders.

The distribution analysis under N treatments showed that all traits followed a normal distribution (Figure S1), which is supportive of a GWAS scan [24]. Compared to the eight founders, the derived WM-800 population showed a wider range of trait variation and a higher coefficient of variation for all studied traits (Table 1 and Figure 1).

This indicates the occurrence of transgressive segregation in population WM-800, which is an important feature to select genetic variation in plant breeding. In total, 20 WM-800 lines outperformed the best elite wheat founder, Tobak, achieving an average yield of 84.93 dt/ha across seven environments and two N treatments. For example, line WM_087 achieved an average yield of 89.33 dt/ha, which is 4.40 dt/ha (+5.2%) above the highest yielding founder Tobak (Table S5). As already indicated for plant height by [44], transgressive segregation can be found in population WM-800 demonstrating its ability to deliver a wide range of phenotypic variation that may be applied to map new loci/genes controlling important yield-related traits.

For all traits studied, the ANOVA indicated highly significant differences at *p* < 0.001 between N treatments for both population WM-800 and the founders, with the exception of GNE among founders with *p* < 0.01 (Table S6). We found that average trait values for both WM-800 and founders always increased under N1, with the exception of TGW, where N0 had higher values (Table S4). These results coincided with other reports, which discovered higher grain yields and delayed development with increased N levels [26,45], while others found that TGW values were increased under reduced N levels [12,19,22]. In general, higher grain protein content can be achieved with an increased N level at the expense of a decreased starch content in grains [46]. Hence, we assume that grain weight is increased under low N levels due to the reverse effect of an increased starch content as it is the main content (~80%) of the wheat grain endosperm [47] and thus has the highest impact on grain weight. Further studies need to be carried out to clarify this assumption.

There was a significant difference among the environments in regard to climate conditions. The growing season of 2017/2018 was much dryer and warmer than 2016/2017, especially in the center of Germany (Hadmersleben and Halle, Table S2), which was one of the warmest and driest seasons since the beginning of regular weather records in Germany [48,49]. Trait heritabilities in WM-800 were generally high with values between 50.1% (MAT under N0) and 96.0% (HEI under N1) except for EAR with values down to 31.3% under N0 (Table S7). This is in agreement with previous reports [22,50], where moderate to high heritability values were detected for the studied traits. In all cases, trait heritabilities increased under N1 treatment, also indicated by higher genetic variance components (Table S7). Our findings confirm that the studied traits are genetically heritable and predominantly controlled by the influence of genotype and less affected by the environment [15,26], which is a prerequisite for subsequent QTL detection.

Correlation among yield components is important to explain the final yield formation. Under both N treatments, Pearson correlation coefficients (r) among the three yield components (TGW, GNE, and EAR) were positively correlated with grain yield and negatively correlated among themselves (Table 2), as reported by [19]. The highest positive correlation was found between HEA and MAT (r_(Across)_ = 0.65) which may indicate a joint control of developmental traits by flowering-related genes, also shown for barley as reported by [51]. In contrast, the highest negative correlation was detected between TGW and GNE (r_(N1)_ = −0.40) which can be attributed to high competition for sink assimilates either producing a high number of grains at a low grain size or vice versa. Monostori et al. found that the negative correlation between TGW and GNE resulted from the complex effect of insufficient N and/or drought stress after anthesis which could lead to high GNE (determined before anthesis) and thus decreased TGW [26]. The observed correlation values indicated that late heading had a positive effect on GNE and increased plant height had a positive effect on TGW under both N treatments with no or minor effects on grain yield. The same trend of correlation was detected between TGW, HEI, and HEA in a European winter wheat collection [52]. The yield was found to be correlated with HEI, TGW, and GNE under both treatments with a maximum of r = 0.31 for YLD*TGW under N1. Based on the correlation analysis, one can expect to detect pleiotropic QTL effects for the studied traits in WM-800.

### 2.2. Characterization of Genetic Diversity in WM-800 Based on SNP Genotyping

The WM-800 population was genotyped by the 15k Infinium SNP array [44] and, in addition, the 135k Affymetrix SNP array which yielded 27,006 polymorphic SNPs (Table A1 of [53]. In total, 11,181, 12,231, and 3594 SNPs were distributed over the wheat A, B, and D genomes, respectively, with a strong bias against the D genome (13.3% of all markers) and a weak bias against the 4A and 4B chromosomes with only 799 and 1226 markers, respectively (Table A2 of [53]. The number of SNPs and the coverage of the D genome is in agreement with previous reports [44,54]. Moreover, [43] reported similar findings presenting a wheat map consisting of a total of 5436 SNP markers for their MAGIC population BMWpop where only 11.9% of the markers were mapped to the D genome (i.e., 647 SNPs).

**Figure 2:**
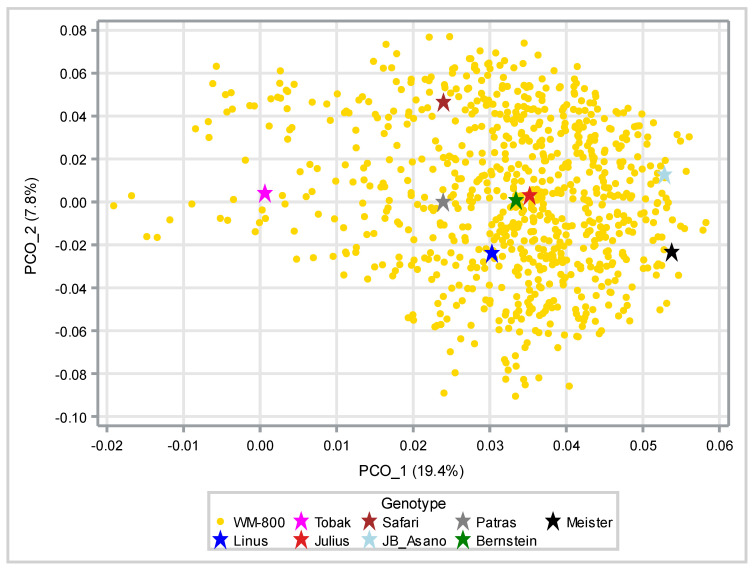
Visualization of the genetic diversity present in WM-800 and its eight founder lines based on a 2-dimensional principal component analysis.

The 27,000 informative SNPs were used to characterize the genetic diversity present in WM-800. For this, genetic similarity among the eight founders and 800 WM-800 lines was estimated (Table C1 of [53]. Among the eight founders, an average genetic similarity of 0.59 was calculated with a range of 0.14 (from 0.53 to 0.66). Among WM-800 lines the diversity was substantially increased with a range of 0.52 (from 0,46 to 0.98) at an average genetic similarity of 0.60. The increase in genetic similarity can also be depicted from the graph made by the first two principal components, explaining 19.4% and 7.8% of the genetic variation based on the genetic similarity values (Figure 2). Here, Tobak and Meister are placed at the opposite ends of the first principle component and Safari and Linus at the opposite ends of the second principle component with 328 WM-800 lines placed outside the area defined by the eight founders (Table C2 of [53]. The WM-800 MAGIC population showed higher genetic diversity than other MAGIC winter wheat populations, for example, the MAGIC wheat population BMWpop, which explained 2.7% and 2.3% of the genetic variation with only a few MAGIC lines laying outside the area defined by the founders [43]. The high degree of diversity present in WM-800 may be attributed to the strong variation of the selected founders in regard to breeder’s origin and wheat quality levels, resulting in a high level of diversity present among the founders and, thus, among the resulting WM-800 lines. Based on the high level of genetic diversity, one may expect to detect a high number of QTL with strong effect variations in WM-800.

Based on SNP estimation of genetic similarities between 800 lines, the first and the second principal components explained 19.4% and 7.8% of the genetic diversity present in WM-800.

### 2.3. Building Haploblocks and Haplotypes in WM-800

We used 27,006 SNPs to build haploblocks (HB) based on LD (Table A1 of [53]. We identified 2970 HBs across 21 wheat chromosomes (Table D2 of [53]. The maximum number of SNPs per haploblock was found for haploblock Chr6D_HB057 consisting of 198 SNPs. Across all 2970 HBs 8498 haplotype (HT) alleles were identified with MAF > 0.05 (Table D3 of [53]. The maximum number of HTs was found for haploblock Chr5D_HB059 with nine HTs, including seven parental HTs and two HTs arising from recombination events in WM-800 [26]. The allelic state of 800 lines of population WM-800 was subsequently deduced for each HT, first, based on an SNP sequence (ACGT) code and, second, based on a 0–1 code for the absence and presence of the respective HT in a particular WM-800 line (Tables D4 and D5, in [53]. In total, 2281 singular SNPs (with 4562 alleles) could not be integrated into any haploblock due to a lack of significant LD with other SNPs. Finally, 8498 HTs and 4562 singular SNP alleles yielded 13,060 markers, which were subsequently used in a GWAS analysis (Table D6 of [53]. Our results suggest that the larger number of SNPs and lines used in WM-800 empowered us to reveal a greater haplotype diversity in comparison to the BMWpop [43]. This finding may indicate that haplotype-based GWAS in WM-800 is an effective approach to detect QTL and estimate the allele-specific QTL effects associated with the studied agronomic traits.

### 2.4. Haplotype-Based GWAS in WM-800

**Figure 3:**
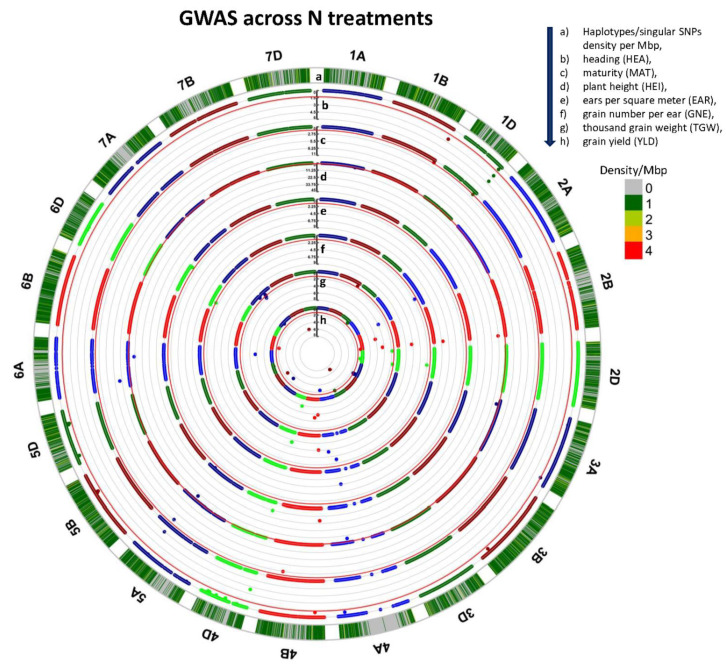
Circular Manhattan plots of genome-wide association scan for seven traits studied in WM-800 across N treatments.

A GWAS based on haplotypes and singular SNPs (the latter not included in haploblocks) was carried out across N treatments and for each N treatment separately to study the genetic regulation of yield and yield components in the MAGIC-WHEAT population WM-800 made of 800 RILs in generation F_4:6_. In total, the GWAS detected 240 significant marker-trait associations (MTAs) with Bonferroni-*p* values (BON_*p*) < 0.05 for seven traits studied across N treatments and under two N treatments (Figure 3 and Figure S2, Table 3 and Table S8). These MTAs are found at 126 haplotypes and 114 singular SNPs (the latter not included in HBs) located on 21 wheat chromosomes. A pivot table showing the significant trait effect estimates per MTA is given per N treatment and across N treatments (Table S9). The total number of detected MTAs was slightly higher for N1 treatment (89 MTAs) than for N0 and across N (71 and 80 MTAs), respectively. In terms of MTAs per trait, TGW showed the highest number with 51 followed by GNE and HEI with 49 and 43 MTAs, respectively, whereas the lowest number was detected for EAR with only 12 MTAs (Table 3). This finding reflects the high heritability found for TGW, HEI, and GNE and the low heritability found for EAR (Table S7). The explained genetic variance ranged from 13.2% for EAR under the N0 treatment to 71.4% for HEI across treatments (Table 3). Compared to SNP-based QTL studies, the explained genetic variance is much higher. This finding may be attributed to the multi-allelic study, which is possible in MAGIC populations using haplotype-based markers. The number of allelic variants, which can be differentiated in a haplotype-based GWAS analysis, for example, eight possible haplotypes per haploblock in an eight-way MAGIC population, is much higher than in a bi-allelic SNP-based study, where only two SNP alleles per locus can be differentiated [55]. The relatively high proportion of explained genetic variance found in HEI and HEA may be indicative of adaptation traits. Scott et al. reported that the strongest QTL effects located for adaptation traits in the NDM MAGIC wheat population “NIAB DIVERSE MAGIC” colocalized with the early flowering allele at the photoperiod locus *Ppd-D1* and the semi-dwarfing alleles at *Rht-B1* or *Rht-D1* [31].

Wheat chromosomes are named on the outside of the circos plot. Circle a denotes the number (density) of haplotypes and singular SNPs per Mbp. The following circles b to h denote the GWAS output across N treatments for heading (HEA), maturity (MAT), plant height (HEI), ears per square meter (EAR), grain number per ear (GNE), thousand grain weight (TGW) and grain yield (YLD). The −log_10_BON_*p* value of each marker-trait association is indicated as a colored dot on the vertical scale bar. For each trait, the red circle indicates the significance threshold at BON_*p* = 0.05 (i.e., −log_10_BON_*p* = 1.301).

In the current WM-800 study, we merged multiple significant MTAs to one QTL, if (1) they were genetically linked within a linkage disequilibrium (LD) window of <5 cM and (2) acted on the same trait, either across or within N treatments. In this regard, the 240 MTAs were compressed to 71 QTL, ranging from 2 to 15 for EAR and GNE, respectively (Table 2 and Table S10). These QTL are present in 137 haploblocks or singular SNPs, i.e., 40, 51, and 46 significant loci found under N0, N1, and across N treatments, respectively. The total number of QTL is lower than the number of significant HBs and singular SNPs since often QTL effects were simultaneously significant under N0, N1, or across N treatments (Table 3 and Table S10). High numbers of QTL were found for GNE, TGW, and YLD with a total of 15, 13, and 12 effects. In contrast, for EAR only 2 QTL effects were detected (Table 3). This may indicate that for GNE, TGW, and YLD more precision in phenotyping was possible and, potentially, more genetic variation was available in WM-800, as already indicated by higher heritability values, compared to EAR (Table S7).

In total, we identified 14 and 25 QTL (i.e., 54.9% of all QTL), which were only detected under one of the two N treatments, either N0 or N1, respectively (Table 3 and Table S10). This finding is, in particular, true for MAT, GNE, and YLD, where 90.0%, 66.6%, and, 58.3% respectively, of the QTL, were either detected under N0 or N1 but not under both N treatments simultaneously. In addition, the number of unique QTL, only present under one N treatment, is substantially higher under N1 than under N0 treatment (25 vs. 14 QTL), which in most cases reflects the higher standard deviation present under N1 than under N0 (Table 1). We, thus, conclude that, to some extent, the genetic regulation of NUE in WM-800 may be different under low and high N availability. In consequence, this finding implies that depending on how much nitrogen from soil and fertilizer sources is available in a target environment, different genes and QTL alleles need to be selected in a wheat breeding program. In addition, 7 out of 71 QTL (9.9%), were exclusively detected in studies across both N treatments. Based on the across N GWAS we detected, for example, two beneficial yield QTL on chromosomes 3D and 6B, bearing yield increasing alleles in WM-800 derived from Julius (+3.37 dt/ha) and Meister, Tobak, and Julius (+2.23 dt/ha), respectively (see Table S10). This finding may be attributed to a low detection power for some MTAs under a single N treatment, supporting our strategy to carry out an extra round of GWAS by combining phenotype data across both N treatments.

Using haplotypes and singular SNP alleles enabled us to estimate founder-specific effects at each QTL (Table S10). The specificity of founder effects depends on the number of haplotypes one can differentiate at each haploblock. Per QTL between two and eight haplotypes could be defined. For example, at the grain number QTL QGNE_7A, defined by haploblock Chr7A_HB189, eight parental QTL alleles could be distinguished in WM-800. This enabled us to estimate individual allele effects at QGNE_7A on a range from −1.97 to +3.11 grains per ear for each founder haplotype. In this ideal case, breeders can directly select the best allele out of the eight founder haplotypes to improve the desired target trait. In the case of QGNE_7A, breeders should select the Patras allele in WM-800 to increase the trait by 3.11 grains per square meter. In contrast, based on a bi-allelic SNP marker only two SNP alleles can be distinguished in WM-800 although, most likely, up to eight different founder alleles are present, reducing the chance to select the hidden best allele at a target trait. Furthermore, Refs. [31,43] successfully used haplotypes in their MAGIC wheat association studies to locate QTL and estimate QTL allele effects. For example, Scott et al. concluded that the 16 founders used in the NIAB MAGIC population showed low haplotype diversity [31]. Moreover, they found a high amount of pleiotropic effects, indicating that selection based on QTL may have additional effects on correlated traits one have to consider in breeding. Our results imply that the haplotype-based GWAS carried out in WM-800 is useful to identify a high number of founder-specific QTL effects as well as pleiotropic effects, which can be used in elite wheat breeding.

### 2.5. Pleiotropic QTL in WM-800

Generally, quantitative genetic studies are focused on the main effect of a QTL. However, additional effects on other traits, known as the pleiotropic effects of a QTL, may be crucial for application in molecular breeding. In the current study, the haplotype-based GWAS in WM-800 revealed 13 pleiotropic QTL effects (within windows of 5 cM), which showed effects on multiple traits simultaneously (Tables S8–S10). The pleiotropic QTL were located on chromosomes 2B, 3A, 4A, 4B, 4D, 5A, 6A, 6D, and 7B. The 13 QTL exerted pleiotropic effects on all seven traits studied. At three loci up to four traits were controlled simultaneously. These are Chr2B_SNP_06906, Chr4D_HB006 and Chr6A_HB035 were the traits HEI, EAR, GNE, TGW, the traits HEA, HEI, GNE, TGW, and the traits MAT, HEI, GNE, TGW, respectively, are controlled simultaneously. The potential genetic co-regulation of HEI, GNE, and TGW is also supported by a positive correlation between TGW and HEI (r_across_ = 0.52) and a negative correlation between TGW and GNE (r_across_ = −0.39) supporting a common regulation of these traits by a joint gene action. Examples of joint genetic regulations of traits are manifold and the semi-dwarf genes may be used as a blueprint.

The QTL on chromosomes 4B and 4D map to the well-studied semi-dwarf genes *Rht-B1* (RefSeq 1.1 gene *TraesCS4B02G043100*) and *Rht-D1*. (RefSeq 1.1 gene *TraesCS4D02G040400*). Since the isolation of the DELLA protein gene *GIBBERELLIC ACID INSENSITIVE* (*GAI*) in *Arabidopsis thaliana* [56], its orthologues have been characterized in other plants as well, for example, *d8* in maize, *VvGAI* in grape, *BrRGA* in *Brassica rapa*, *SLN1* in barley, *Rht* in wheat, and *SLR1* in rice [57]. The encoded proteins share an N-terminal DELLA domain, where domain mutations render the proteins resistant to GA-induced degradation resulting in dwarf phenotypes. So far, more than 20 reduced height genes (Rht) and alleles have been reported in wheat, including GA-insensitive genes (*Rht-B1b, Rht-B1c, Rht-B1d, Rht-D1b, Rht-D1c*, etc.) and GA-sensitive genes (*Rht4, 5, 6, 7,* etc.) [57].

The founders of WM-800 carry different dwarfing alleles at the two *Rht* genes (*Rht-B1.b* and *Rht-D1.b,* respectively) as described by Sannemann et al. resulting in contrasting effects on the traits under study (Table S10) [44]. The haploblock Chr4B_HB018 containing *Rht-B1* was associated with effects on three traits (HEI, GNE, and TGW). At this locus, the semi-dwarf containing haplotype Chr4B_HB018_04274, derived from Tobak and Safari, simultaneously decreased HEI by −9.52 cm and TGW by −2.83 g and increased GNE by 3.00 grains per ear under N1 treatment (Tables S8 and S9). In contrast, the alternative haplotype Chr4B_HB018_04273, derived from the remaining six founders of WM-800, increased HEI by 8.01, TGW by 2.57 g, and decreased GNE by −3.13 grains per ear under N1 treatment. Likewise, the QTL on chromosome 4D, containing the stronger semi-dwarf gene *Rht-D1.b,* acted on four traits simultaneously (HEA, HEI, GNE, and TGW). At this locus, the semi-dwarf containing haplotype Chr4D_HB006_04687, derived from Patras, Linus, JB Asano, and Julius, decreased HEI by −9.65 cm, TGW by −2.48 g and increased HEA by 0.83 days and GNE by 3.53 grains per ear under N1 treatment. The alternative haplotype Chr4D_HB006_04688, derived from the remaining four founders, exhibited exactly the opposite trait effects. Pleiotropic effects of *Rht-B1* and *Rht-D1* have been described in other reports as well [58,59,60,61,62,63]. Our findings, thus, confirm previous studies. Plant height variation in a set of European winter wheat cultivars was also highly influenced by *Rht-B1* and *Rht-D1* [64]. *Rht-B1* was found to control plant height, grain yield, quality, and the plant’s adaptability to N-deficient environments [22]. It was also reported that *Rht-B1* is associated with nitrogen uptake efficiency (NUpE) and nitrogen utilization efficiency (NUtE) [14]. In addition, pleiotropic effects of *Rht-B1b* and *Rht-D1b* on plant height and heading date were detected in a worldwide winter wheat collection by [65] and of *Rht-D1* in a soft-red winter wheat RIL population by [66]. However, in WM-800 the *Rht* genes were not associated with significant effects on yield, although both genes were known to increase grain yield during the green revolution [59]. This may be due to the fact that all eight founders of WM-800 are high-yielding recent elite cultivars derived from the modern winter wheat breeding pool, where wild-type *Rht* effects may be balanced by background genes, which were selected for high yielding past the green revolution. We, thus, conclude, that the *Rht* genes present in the elite founder cultivars may not be useful to further increase yield in population WM-800.

A further QTL showing pleiotropic effects was located at the end of chromosome 2B (Tables S8–S10). The singular SNP Chr2B_SNP_06906 was associated with four traits (HEI, EAR, GNE, and TGW). At this locus, the SNP allele Chr2B_SNP_06906_G, derived from six founders of WM-800, increased HEI by 4.28 cm, GNE by 2.31 grains per ear, TGW by 1.67 g and decreased EAR by −28.14 ears per sqm under N1 treatment. The alternative SNP allele Chr2B_SNP_06906_G, derived from Bernstein and Julius, exhibited the opposite effects. The physical position of the significant SNP is in close proximity to members of a gene family coding for a *NITRATE TRANSPORTER 1 (NRT1)/PEPTIDE FAMILY (NPF*) [67]. The *NRT1/PTR* genes are known for the uptake and translocation of nitrates and small peptides in higher plants. In wheat, the *NPF* gene family comprises a total of 331 *NPF* genes, predominantly located at the proximal end of the long arm of chromosomes. Among the eight *NPF* subfamilies of wheat, *NPF5* is the largest subfamily including 34 members [67]. An overexpression study of the rice gene *OsPTR9* revealed an increased grain yield due to an enhanced ammonium uptake as well as advanced lateral root formation [68]. The closest member of the NPF family, *TraesCS2B02G615500* coding for *TaNPF5.34,* is only 1.07 Mb upstream of Chr2B_SNP_06906 [69]. *TaNPF5.34* is known as a low-affinity nitrate transporter that may explain the positive effects associated with the SNP allele Chr2B_SNP_06906_G on GNE and TGW under N1 treatment. In WM-800, *TaNPF5.34* may be a favorable candidate gene to increase GNE and TGW, however, reducing EAR at the same time, with no significant effect on YLD. It has also been shown, that overexpression of the rice *NITRATE TRANSPORTER* gene *OsNRT2.3b,* a homolog of *TaNPF5.34*, increased grain yield and nitrogen use efficiency by 40% in field trials. [70]. According to Wang, et al., the transcript *TaNPF5.30,* located next to *TaNPF5.34,* was highly expressed in roots under high N supply indicating that this gene might be involved in nitrate reallocation between tissues and in controlling the nitrate balance between cell compartments as a response to N flux [67].

Also, a QTL on chromosome 4A showed pleiotropic effects on three traits (Tables S8–S10). At this locus, the SNP allele Chr4A_SNP_12125_A, derived from Patras, Meister, Linus, and Safari, increased EAR by 24.87 ears per sqm and decreased GNE by −2.24 grains per ear under N0 and N1 treatment, respectively. The neighboring SNP allele Chr4A_SNP_12131_A, derived from the same founders as Chr4A_SNP_12125_A, was genetically placed only 0.16 cM distant to Chr4A_SNP_12125, although physically separated by 51.0 Mb. The former allele was associated with an HEI decreased by −3.78 cm under N1 treatment. A further member of the *NITRATE TRANSPORTER* gene family, *TaNPF4.9* (*TraesCS4A02G225400*) is located 9.34 Mb downstream of Chr4A_SNP_12131 [67,69]. The increased EAR, in particular under N1 treatment, may be attributed to the *NITRATE TRANSPORTER TaNPF4.9*. Efficient allocation of nutrients and suitable source-sink interactions are crucial during the spike and reproductive development [71], therefore, higher N fertilization could improve agronomic traits including EAR per square meter.

We conclude that the definition of pleiotropic QTL effects is helpful to explain the parallel effects of genes or QTL on independent physiological traits, which may be visible as significant correlations between the involved traits. In addition, the definition of pleiotropic QTL may support plant breeding since the occurrence of negative correlations between desired agronomic traits may be attributed either to pleiotropy or to genetic linkage. The pleiotropic effects of a single gene cannot be resolved by classical breeding. However, the linkage between two independent genes can be broken by selecting recombinations. Endogenous genes shaping the genome-wide recombination landscape, for example, the barley meiosis-specific *α-KLEISIN COHESIN SUBUNIT* gene *REC8*, may be a genetic tool used in future breeding programs to increase the recombination rate in cereals, supportive to identifying rare recombination events and to break an unfavorable link between two neighboring alleles [72].

### 2.6. QTL, Presented Separately for Each Trait in WM-800

In the following, significant QTL identified by GWAS based on haploblocks and singular SNPs are presented separately for each of the seven traits studied in WM-800.

#### 2.6.1. QTL Controlling Heading (HEA)

In total, nine QTL, derived from 27 MTAs, were associated with HEA across N (6), under N0 (4) and N1 (7) treatments (Table 3 and Tables S8–S10). HEA did not show high variation, most likely due to the selection of modern winter wheat elite cultivars as founders for the WM-800 population, which showed little variation in flowering time (CV = 1.2% within WM-800, Table 1). Nevertheless, the haplotype Chr1D_HB070_01194 on chromosome 1D was associated with HEA. The allele effect derived from Patras, Linus, and Safari increased HEA by a maximum of 0.75 days under N0 treatment. Heading, respectively flowering time in wheat is mainly determined by vernalization genes (*Vrn*) and photoperiod response genes (*Ppd*), and, in addition, by EARLINESS PER SE genes (*Eps*), where *Eps* genes correspond to gene effects not controlled by *Vrn* and *Ppd* genes [73]. We did not identify the effects of the *Vrn* and *Ppd* genes on HEA. This may be due since those genes are assumed to be adapted to regional growth conditions, allowing only minor genetic variation to be present in a regional gene pool. However, the *Eps* gene *EARLY FLOWERING 3* (*TaELF3-1DL)* was reported to control heading date in wheat [74]. We found *TaELF3-1DL* (*TraesCS1D02G451200)* only 1.61 Mb upstream of Chr1D_HB070_01194 [69], which may explain the HEA effect. The strongest QTL effect on HEA was detected at haploblock Chr5A_HB092. The haplotype Chr5A_HB092_05026, derived from Bernstein, was associated with an increase in HEA by 1.57 days under N1 treatment. It remains open if the flowering time-related genes, *PhyC (TraesCS5A02G391300)* or *VRN-A1* (TraesCS5A01G391700) located approx. 40 Mb downstream on chromosome 5A are responsible for the reported HEA effect [69].

#### 2.6.2. QTL Controlling Maturity (MAT)

Only ten QTL, derived from 24 MTAs, were associated with MAT across N (3), under N0 (6) and N1 (5) treatments (Table 3 and Tables S8–S10). Out of those, a high number of nine QTL (90%) were solely detected under N0 (5) or N1 (4) treatments, indicating different genetic regulations of MAT under low and high N availability. In similarity to HEA, MAT showed little variation (CV = 0.9% within WM-800, Table 1). One increasing QTL effect was located on chromosome 1B. The haplotype Chr1B_HB242_00987, derived from Patras, Meister, and JB Asano, delayed maturity by 0.67 days under N0 treatment. The same trend was seen for HEA, which is in accordance with the considerably high correlation found between both traits (r = 0.65). Interestingly, the *EARLY FLOWERING 3* gene (*TaELF3)* is located only 1.75 Mb upstream of Chr1B_HB242_00987. This gene is known to enhance flowering time in wheat and is the candidate gene for *Eps-B1* [75]. In contrast, the haplotype Chr6A_HB035_06101, derived from Patras, JB Asano, and Bernstein, was associated with a maturity −1.13 days earlier under N1 treatment. Most likely, this QTL effect can be attributed to the *NAC TRANSCRIPTION FACTOR* gene *Nam-A1* (*TraesCS6A02G108300)*, located only 10.20 Mb upstream, which was reported to control senescence in wheat [76,77]. An Australian field trial with 19 cultivars reported a negative correlation between senescence and grain yield, and between harvest index and nitrogen use efficiency, due to the avoidance of unfavorable, hot, and dry, summer conditions [78]. In this case, high yielding was explained by a pleiotropic effect of the *NAM-A1* gene controlling senescence under Western Australian conditions. In our field study, we did not see the pleiotropic effects of the *NAM-A1* gene on yield or other yield-related traits. The yield effect of *NAM-A1* may, thus, require more severe drought conditions as one can find, for example, in Australia.

#### 2.6.3. QTL Controlling Plant Height (HEI)

Altogether, ten QTL, derived from 43 MTAs, were associated with HEI across N (7), under N0 (9) and N1 (8) treatments (Table 3 and Tables S8–S10). The strongest height effect was located at the semi-dwarf gene *Rht-D1* on chromosome 4D. The haplotype Chr4D_HB006_04687, derived from Patras, Linus, and JB Asano Julius, reduced plant height by −9.65 cm under N1 treatment. The second strongest height effect was located at the semi-dwarf gene *Rht-B1* on chromosome 4B. The haplotype Chr4B_HB018_04274, derived from Tobak and Safari, reduced plant height by a maximum of −9.52 cm under the N1 treatment. For *Rht-B1* and *Rht-D1*, pleiotropic effects on HEI, GNE, and TGW, respectively on HEA, HEI, GNE, and TGW have been described above and confirm a number of earlier reports cited above. In addition to the semi-dwarf genes, two further height QTLs were detected in haplotypes on chromosomes 5A and 6A. The haplotype Chr5A_HB081_04991, exclusively derived from Patras, increased plant height by 5.45 cm under N1 treatment. The haplotype Chr6A_HB059_06151, derived from Patras, Meister, Linus, Tobak, and Safari reduced plant height by −4.30 cm under N1 treatment. Only 1.36 Mb downstream of this QTL the gene *TraesCS6A02G221900* is located [69]. Recently, this gene was cloned by [79] as the *Rht24* gene acting as a *GIBBERELLIN (GA) 2-OXIDASE (TaGA2ox-A9).* This gene may explain the HEI effect of Chr6A_HB059_06151. So far, more than 20 *Rht* genes are recorded [80]. However, only a few *Rht* genes that reduce height without adverse effects on yield are used in wheat breeding programs. These include the gibberellin-insensitive semi-dwarfing alleles *Rht-B1b* and *Rht-D1b* and the gibberellin-responsive allele *Rht24b*, which are globally present in elite wheat cultivars since the Green Revolution. In particular, *Rht24b* and *Rht-D1b* are prevalent in wheat breeding programs, being present in 67 and 49% of a global collection of wheat cultivars released after 1990 [81]. Interestingly, the reduced height allele of *Rht24* was already present in the wheat breeding pool before 1960, prior to the Green Revolution. Whereas *Rht-B1b* and *Rht-D1b* lose their harvest index and yield advantage in lower fertility environments [81,82], the *Rht24b* allele shows no or at least less adverse effects on yield under a broad range of climatic conditions. This may indicate that the *Rht24b* allele should be used in breeding programs, in particular, under climate change scenarios.

#### 2.6.4. QTL Controlling Ears Per Square Meter (EAR)

Only two QTL, derived from 12 MTAs, were associated with EAR across N (2), under N0 (2) and N1 (2) treatments (Table 3 and Tables S8–S10). The strongest EAR effect was located at the singular SNP allele Chr2B_SNP_06906_T, derived from the founders Bernstein and Julius. The allele increased EAR by 28.14 ears per square meter under N1 treatment. This effect may be attributed to the *NITRATE TRANSPORTER 1 (NRT1)/PEPTIDE FAMILY* gene *TaNPF5.34* (TraesCS2B02G615500), which is located only 1.07 Mb upstream of the SNP [67]. A second EAR-improving effect in WM-800 was detected on chromosome 4A. The singular SNP allele Chr4A_SNP_12125_A, derived from Patras, Meister, Linus, and Safari, increased EAR by 24.87 ears per square meter under N1 treatment. It remains open, which gene is responsible for the trait-improving effects on chromosome 4A.

#### 2.6.5. QTL Controlling Grain Numbers Per Ear (GNE)

Altogether, 15 QTL, derived from 49 MTAs, were associated with GNE across N (8), under N0 (7) and N1 (12) treatments (Table 3 and Tables S8–S10). This is the highest number of QTL detected for any of the traits studied in WM-800. Out of those, a high number of ten QTL (66.6%) were solely detected under N0 (3) or N1 (7) treatments, indicating different genetic regulations of GNE under low and high N availability. Among the strongest GNE effects were the two semi-dwarf genes *Rht-B1* and *Rht-D1* on chromosomes 4B and 4D, respectively. The haplotype Chr4B_HB018_04274, derived from Tobak and Safari, increased GNE by 3.00 grains per ear under the N1 treatment. The haplotype Chr4D_HB006_04687, derived from Patras, Linus, JB Asano, and Julius, increased GNE by 3.53 grains per ear under the N1 treatment. The pleiotropic effects of *Rht-B1* and *Rht-D1* on HEI, TGW, and GNE were already discussed before well [58,59,60,61,62,63].

#### 2.6.6. QTL Controlling Thousand-Grain Weight (TGW)

Altogether, 13 QTL, derived from 51 MTAs, were associated with TGW across N (12), under N0 (7) and N1 (10) treatments (Table 3 and Tables S8–S10). In WM-800, most QTL showed low effects on TGW. The strongest TGW effect was located at the semi-dwarf locus Chr4B_HB018. The non-semi-dwarf allele Chr4B_HB018_04273, derived from Patras Meister, Linus, JB Asano, Bernstein, and Julius, increased TGW by 2.57 g. Likewise, the non-semi-dwarf allele Chr4D_HB006_04688, derived from Meister, Tobak, Bernstein, and Safari increased TGW by 2.48 g under N1 treatment. Both findings are in agreement with the overall positive correlation of r = 0.52 between HEI and TGW (Table 2). The grain weight-increasing effects of the wild-type *Rht-B1a and Rht-D1a* alleles are also in agreement with [83] but in contrast to [84]. The former study was conducted with 225 DH lines developed from a cross between Westonia and Kauz tested in Western Australia and the latter with 95 soft red winter wheat doubled haploid (DH) lines in Arkansas, USA. This finding indicates that the *Rht-D1* effect may depend on the genetic background of the studied genotypes and the environmental conditions, which are present at the test site.

A further positive TGW effect was located on chromosome 6A. The haplotype Chr6A_SNP_19718_T, derived from JB Asano, Bernstein, and Julius, increased TGW by a maximum of 1.62 g under N1 treatment. Possibly, this effect can be attributed to the semi-dwarf gene *Rht24 (TaGA2ox-A, TraesCS6A02G221900),* which is located 15.24 Mb upstream of the haplotype and reported to control grain weight [80]. Many TGW QTL have been described on almost all wheat chromosomes, for example, [21,85,86,87], but only those candidate genes mentioned before are close to the QTL found in WM-800. Interestingly, also the gene controlling grain protein content *(GPC-B1,* i.e., *Nam-B1)* on chromosome 6B, was reported to exert pleiotropic effects on grain weight, in addition to senescence and Fe and Zn content in wheat grains. However, we could not find a TGW QTL in WM-800 neither near this gene nor near its homoeologs *Nam-A1* (i.e., *TraesCS6A02G108300* at position 77,097,709–77,100,361) on chromosome 6A and *Nam-D1* (i.e., *TraesCS6D02G096300* at position 60,486,167–60,488,033) on chromosome 6D [76,77].

#### 2.6.7. QTL Controlling Grain Yield (YLD)

Grain yield is determined by three main yield components: i.e., ears per square meter, grain number per ear, and grain weight. The three yield components were tested in three, four, and seven environments, respectively (Table 5). Grain yield is known to be under polygenetic control and to highly depend on environmental conditions. Nevertheless, improving grain yield is still the most important breeding goal, potentially winning further attraction under climate change scenarios, where the focus may be laid on yield stability under varying abiotic and biotic stress conditions including low N supply as tested in our study. Altogether, 12 QTL, derived from 34 MTAs, were associated with YLD across N (8), under N0 (5) and N1 (7) treatments (Table 3 and Tables S8–S10). Out of those, a high number of seven QTL (58.3%) were solely detected under N0 (2) or N1 (5) treatments, indicating different genetic regulations of YLD under low and high N availability. At nine loci significant (BON_*p* < 0.05) QTL effects increasing yield between 2.00 and 4.47 dt/ha could be detected. The strongest YLD-increasing effects were located on chromosomes 3A and 7B. The haplotype Chr3A_HB130_02953, derived from Julius, and the haplotype Chr7B_HB035_07909, derived from Tobak and Safari increased YLD by 4.47 and 4.23 dt/ha, respectively. Both QTLs could not be related to candidate genes or to previously reported QTL effects. They may embody novel yield QTL, which potentially entered the named founder genomes due to phenotypic selection without prior notice of their existence as a QTL. It also remains open, if the favorable yield haplotypes were selected in wheat breeding programs due to their yield-increasing effects or due to pleiotropic effects on undisclosed additional traits. This characteristic may hold true for most of the yield-increasing QTL effects found in WM-800 (Table S10). However, QTL effects on chromosomes 3A, 3B, 3D, and 5A may be attributed to the action of the gibberellic acid-related genes *GA20ox 2, GA2bdox,* and *Rht12,* respectively [69,88,89]. These loci were associated with yield-increasing effects of 3.32, 2.74, 3.37 and 2.71 dt/ha across N treatments in case the haplotypes derived from Julius (3A), JB Asano, Tobak and Safari (3B), Julius (3D) and JB Asano and Bernstein (5A) were present in WM-800 lines, respectively.

Compared to classical QTL studies, based on bi-parental populations or association panels, the number of nine yield-increasing QTL effects appears relatively high [12,90,91,92,93,94,95,96,97,98,99]. Compared to DH populations, the high number of eight founders rather than two, the number of five meiotic generations rather than one, and the large population size of N = 800 of the MAGIC-WHEAT population rather than N < 200 may be attributed to causing the high numbers of segregating QTL alleles and recombination events in WM-800. This in turn may have resulted in a high level of trait variation and a high chance to identify trait-improving QTL effects in the MAGIC population WM-800.

Interestingly, the main semi-dwarf genes *Rht-B1*, *Rht-D1,* and *Rht24* on chromosomes 4B, 4D, and 6A, respectively, showed no effect on YLD in WM-800, neither under N0 nor under N1 treatments, although affecting plant height and other traits studied. As said before, we conclude that the *Rht* genes present in the elite founder cultivars may not be useful to further increase yield in population WM-800. It is worth noting that at the nine yield-increasing QTL mentioned before, the founders JB Asano, Tobak, and Safari contributed five positive alleles to WM-800 whereas the other founders contributed only one (Meister and Bernstein) two (Patras) or three (Linus and Julius) positive alleles. This finding is well reflected by the yield performance of the founders themselves, where Tobak and Safari range highest in yield across N treatments with 84.93 and 83.88 dt/ha, respectively, and Bernstein lowest with 75.73 dt/ha (Table S4).

**Figure 4:**
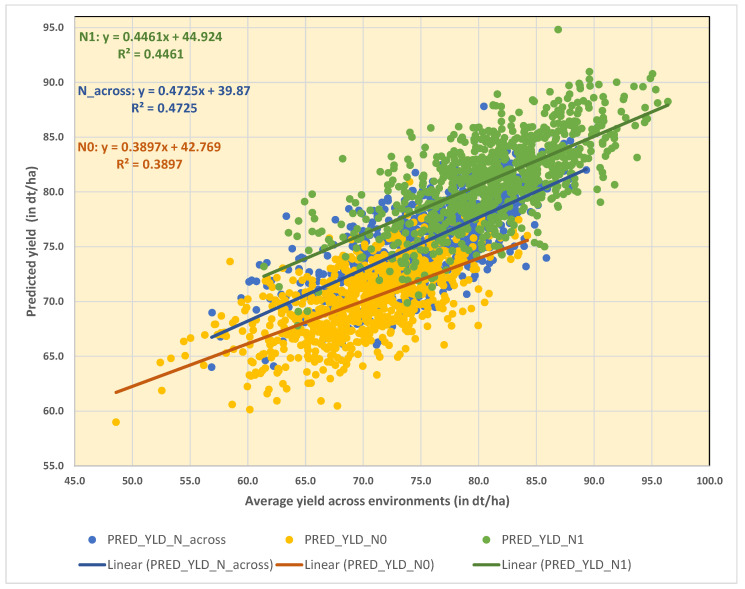
Multiple linear regression plot of predicted yield by actual yield averaged across N treatments and within N0 and N1 environments, respectively. The stepwise regression procedure HPREG selected 9–10 informative QTL alleles and 3 informative quantitative traits for yield prediction.

We conducted a multiple regression analysis including 23 significant haplotypes and singular SNP alleles at twelve yield QTL plus the six agronomic and developmental traits listed in Table 5 using the stepwise regression procedure HPREG implemented in SAS. We found that 9–10 informative QTL alleles and the indicative yield components TGW, GNE, and EAR passed the Schwarz Bayesian information criterion (SBC) and were selected to predict yield (Table S11). The selected 9–10 QTL alleles and three yield components explained less than half of the yield variance present in WM-800 and the eight founders with R^2^ values equivalent to 39.0, 44.6, and 47.3% under N0, N1, and across N treatments, respectively (Figure 4, Table S12). Without the three yield components, the R^2^ values dropped to 25.9%, 26.8%, and 29.7% under N0, N1, and across N treatments, respectively (data not shown). These results indicate that (1) the yield prediction ability is best across N treatment but inferior under N1 and the stress treatment N0. (2) Slightly more than half of the yield prediction ability was achieved based on the 9–10 informative QTL alleles and the remaining part could be attributed to the three yield components TGW, GNE, and EAR.

The findings, reported here, are currently applied in running wheat breeding programs. For this, yield improvements may be achieved by backcrossing favorable WM-800 lines with elite winter wheat genotypes and selecting offspring carrying the identified yield-improving QTL alleles of this study. This process may be accompanied by early generation yield prediction based on the presence of the yield-improving QTL alleles and estimating of the indicative three yield components TGW, GNE, and EAR. Elaborated concepts for haplotype-based selection in breeding programs are available, for example [100,101,102,103,104].

## 3. Material and Methods

### 3.1. Plant Material

The winter wheat MAGIC-WHEAT population WM-800 is based on eight modern German elite cultivars Patras, Meister, Linus, JB Asano, Tobak, Bernstein, Safari, and Julius (hereafter called founders), which vary according to yield, quality, and resistance traits. The eight founders were derived from six breeders and have been released between 2008 and 2017 (Table S1). The eight-way crossing scheme of the WM-800 population was adapted from [37] and resulted in a set of 800 recombinant inbred lines (RILs) in generation F_4:6_. Detailed information about the development of the multi-parental WM-800 population is given in [44].

### 3.2. Field Trials

The WM-800 population was cultivated under field conditions at four locations in Germany. These are: Hadmersleben (Syngenta Seeds GmbH; 51°98′29.07′′ N, 11°29′93.28′′ E; hereafter abbreviated as HAD), Halle (Martin-Luther-University Halle-Wittenberg, 51°29′37.2′′ N, 11°59′20.0′′ E; HAL), Moosburg (Secobra Saatzucht GmbH; 48°28′47.4′′ N, 11°54′30.0′′ E; MOS) and Seligenstadt (KWS LOCHOW GmbH; 49°51′16.3′′ N, 10°06′02.3′′ E; SEL). Plant cultivation took place during the growing seasons 2016/2017 and 2017/2018, resulting in seven field environments (i.e., location × year combinations), excluding Moosburg 2016/17, where no yield trials were conducted. Informations about locations and climate conditions are given in Table S2.

The experiments included two contrasting nitrogen fertilization levels per location. These are standard nitrogen fertilization (**N1**) and stress-inducing low nitrogen fertilization (**N0**). The difference between the two nitrogen levels ranged between 130 and 145 kg N ha^−1^, except for Hadmersleben 2017 with an accidentally low difference of only 40 kgN/ha between the N levels (Table 4). The amount of N fertilizer was added to the field using a fertilizer spreader. The field design was based on randomized complete blocks made of 848 plots for each N treatment, where each block was surrounded by a standard winter wheat cultivar to reduce edge and positional effects. In each environment, the eight founders and four additional local cultivars were replicated four times under each nitrogen treatment. The 800 WM lines were grown in one replication per treatment and environment. Field and disease management was carried out in accordance with local practice. Details on the experimental field trials are given in Table 4.

### 3.3. Phenotypic Data

The phenotypic data of WM lines and founders were measured under two N treatments in seven environments (Table S3). A summary of all investigated traits including the method of their measurement and the number of environments investigated is given in Table 5.

### 3.4. Statistical Analyses

All statistical analyses were carried out using SAS Enterprise Guide 8.3 (SAS Institute Inc., Cary, NC, USA). The analysis of variance (ANOVA) was calculated with *PROC GLIMMIX*. We assumed genotype, environment, and treatment as fixed main effects and included all possible two-fold interaction effects. For each genotype, the least-squares means (LSMEANS) were determined across environments and N treatments as well as per environment and per N treatment.

Variance components were estimated by modeling the random factors genotype, environment, and treatment as well as the corresponding two-way interactions with *PROC VARCOMP*. Broad sense heritabilities (h^2^) of traits were calculated for WM-800 [1] and founders [2] separately based on genotype LSMEANS per environment and N treatment as follows:

(1) $$\;h^{2}=\frac{V_{G}}{V_{G}+\;\frac{V_{GE}}{e}+\frac{V_{GT}}{t}+\frac{V_{R}}{e*t}}$$

(2) $$\;h^{2}=\frac{V_{G}}{V_{G}+\;\frac{V_{GE}}{e}+\frac{V_{GT}}{t}+\frac{V_{GET}}{e*t}+\frac{V_{R}}{e*t*r}}$$

*V_G_*, *V_GE_*, *V_GT_*, *V_GET,_* and *V_R_* correspond to the variance components genotype, genotype*environment, genotype*N treatment, genotype*environment*N treatment, and residual variance, respectively. The variables e, t, and r represent the number of tested environments (between 2 and 7, see Table 4), N treatments (2), and replicates within each environment*N treatment combination (4, only for founders), respectively. Figures were made using RStudio [106] with the package ggplot2 [107].

### 3.5. Genotype Data

The 800 genotypes of WM-800 were genetically characterized by two SNP arrays: (1) a 15k Infinium SNP array containing 13,006 SNPs, where genotyping and SNP data processing was already described in [44]. In addition, (2) the WM-800 lines were genotyped with a 135k Affymetrix SNP array containing 136,780 SNPs designed by TraitGenetics, Gatersleben (http://www.traitgenetics.com), a subsidiary of SGS Institut Fresenius GmbH, Taunusstein, Germany. For this, bulked DNA from twelve seedlings per WM-800 line in F4:5 generation or per founder cultivar was extracted and subjected to SNP genotyping at TraitGenetics.

Subsequently, SNPs polymorphic in WM-800 and with SNP calls for all eight founders underwent a quality check. Only SNPs with (i) <5% missing calls in population WM-800, (ii) a minor allele frequency MAF > 5% and (iii) a known physical position in the wheat genome were kept. Chromosomal positions and physical base pair positions, anchored to the IWGS RefSeq 1.1 reference genome sequence of Chinese spring [69] were provided by TraitGenetics for both the Affymetrix array and the Infinium array [44]. The position of SNPs can be viewed with the genome browser at https://urgi.versailles.inra.fr/jbrowseiwgsc. The merging of both arrays resulted in a total of 27,006 polymorphic and physically anchored SNPs, including 19,522 SNPs of the 135k Affymetrix array and 7484 SNPs of the 15k Infinium array (given in Tables A1 and A2 in [53]. In addition, 7245 SNPs of the Infinium array were assigned to genetic cM positions on wheat chromosomes based on the wheat consensus map [44,108]. The Affymetrix SNPs, without genetic cM positions, were assigned to the wheat consensus map by placing an unmapped SNP between the two closest mapped markers based on the physical SNP position given by [69], Table A1 of [53].

To carry out subsequent regression analyses, the original SNP genotype code (A,C,G,T) was transcribed into a numerical code (0,1,2) based on the presence of the Julius founder allele. At each SNP, WM-800 lines were assigned the SNP values 2 and 0 if the respective SNP genotype contained two Julius alleles (i.e., homozygous Julius) or two Non-Julius alleles (i.e., homozygous Non-Julius), respectively. Heterozygous genotypes, containing one Julius and one Non-Julius allele, were assigned the SNP value 1 (Table B1 of [53]. Missing SNP calls were predicted by applying the mean imputation (MNI) approach [109]. Julius was selected as the reference because the Julius full genome is available [110] and a BLAST database of wheat genome assemblies, including Julius, can be queried at https://galaxy-web.ipk-gatersleben.de/.

Haploblocks (HB), which are made of SNPs in high linkage disequilibrium, were built using the software package Haploview 4.2 [111]. For this, the available set of SNP genotypes of the eight WM-800 founders (Table A1 of [53] was selected to build HBs using Haploview’s ‘Four Gamete Rule’ based on SNPs with a minor allele frequency MAF > 0.05. For each SNP pair with a distance of <500 kb, a haploblock was formed and extended by consecutive SNPs if (i) at least one out of the four possible gametes was observed with a frequency of <0.01 and (ii) a strong LD was estimated between the SNP pair with D’ = 1.0. SNPs not included in HBs were kept as so-called ‘singular SNPs’.

The physical position (in bp) and genetic position (in cM) of the used 27,006 SNPs are given in Table D1 of [53]. In total, Haploview identified 2970 HBs, representing between 2 and 198 SNPs (mean = 8.3; Table D2 of [53]. Across all HBs a total of 92,734 HTs were identified in WM-800. Out of those, 8498 informative HTs were selected passing the quality criteria of (i) HT frequency of >5% in WM-800 and (ii) HT sequence without missing nucleotides (Table D3 of [53]. A genotype matrix containing the genotype scores for 800 WM lines and eight founders at the selected 8498 HTs is given in Table D4 of [53]. Finally, the numerical genotype scores of the selected 8498 HTs and 4562 singular SNPs (i.e., SNPs not included in HBs) were coded as 0 (absent) or 1 (present) for each WM-800 line in Tables D5 and D6 in [53] containing only HTs and merged HTs and singular SNPs, respectively.

### 3.6. Estimating Genetic Similarity in WM-800

The simple matching SAS procedure *PROC DISTANCE* was applied to Table A1 of [53] to calculate genetic similarity estimates between WM-800 lines and the eight WM founder cultivars (Table C1 of [53]). Based on genetic similarity estimates, the population structure was characterized by applying a principal component analysis (PCA) with SAS procedure *PROC PRINCOMP* (Table C2 of [53]).

### 3.7. Genome-Wide Association Mapping (GWAS) Based on Haplotypes and Singular SNPs

The estimation of a marker-trait-association (MTA) based on the calculation of trait least squares means (LSMEANS) for each WM-800 line per N treatment or across N treatments followed a robust three-step procedure performed in SAS 9.4 (SAS Institute Inc., Cary, NC, USA). Beforehand, the genotype set (Table D6 of [53], comprising 8498 haplotypes (HT) and 4562 singular SNPs, i.e., 13.060 markers, which are numerically coded with 0 and 1 for absence and, respectively, presence in a particular WM-800 line, and the phenotype set (Table S4), comprising the trait LSMEANS across N treatments and per N treatment for each WM-800, were merged. The following steps 1 and 2 were executed to select a set of markers as cofactors for the final GWAS in step 3. (1) In step 1, the genotype and phenotype data set was subjected to a cross-validated multiple linear regression run with PROC GLMSELECT in order to identify potentially associated markers based on stepwise forward and backward selection. For this purpose, 80% of phenotype data were randomly assigned to the prediction set and 20% were assigned to the validation set. Markers were consecutively included in and removed from the final model as long as they were able to decrease the average square error (ASE) of phenotype prediction in the validation set. This procedure was repeated 100 times with independent random assignments and the number of times a marker was included in the final model was recorded. Only those haplotypes and singular SNPs that were included in more than one out of 100 models were treated as robust and included in the second step. (2) In step 2, a second, non-repeated multiple linear regression was executed with PROC GLMSELECT restricted to the set of robust and cross-validated markers selected in step 1. Here, the complete phenotype data set was applied to a multiple linear regression run with a stepwise forward selection of markers based on minimizing the Schwarz Bayesian Criterion (SBC, [112]. The final list of selected markers, hereafter called cofactors, was used in the next step. (3) In step 3, each of the 13,060 markers was finally tested for significance by multiple linear regression analysis including the set of selected cofactors modeled in the background with PROC REG. By applying the model option PARTIALR2 (SEQTESTS), marker effects, R², and *p*-values were estimated as a function of the cofactors, which entered the model according to their ranking in step 2. As marker effects were estimated based on quantitative presence-absence of genotype scores (1–0), founder effects could be estimated directly from marker effects, based on the genetic constitution of each founder at the respective marker locus. Significant MTAs were defined at a Bonferroni-Holm [113] adjusted *p*-value of BON_*p* = 0.05. MTAs were joined to one QTL if they were in linkage disequilibrium (LD) with r^2^ ≥ 0.8 according to [114]. This threshold corresponded to a genetic distance of 5 cM, which was already applied in WM-800 by [44].

## 4. Conclusions

Considering the current restrictions in nitrogen fertilizer management in European countries, new sustainable winter wheat cultivars need to be selected to achieve higher yields with less fertilizer input. The MAGIC wheat population WM-800 was grown in a total of seven environments during two seasons in Germany under two contrasting nitrogen (N) treatments in order to characterize its genetic yield potential and to select improved breeding lines. WM-800 lines showed highly significant differences between the two nitrogen treatments, as well as high variances for the seven agronomic traits studied. The highest yielding WM line, WM_087, achieved a yield of 89.33 dt/ha averaged across seven environments and two N treatments, which is an improvement of 4.40 dt/ha (5.2%) compared to Tobak, the best founder cultivar of WM-800. This finding proves that multi-parental populations are useful to increase variability and to find high-performing offspring genotypes for elite breeding.

A genome-wide association study (GWAS), based on 8498 haplotypes and 4562 singular SNPs, located QTL for all yield and growth-related traits studied. In total, 40 and 51 QTL were estimated under low and high N treatments, respectively, and 46 QTL were detected across both N treatments. The haplotype-based GWAS was allowed to estimate the trait effects for individual founder alleles in WM-800, improving the accuracy of QTL detection. For example, the effect of QYLD_3A could be associated with the haplotype allele of cultivar Julius increasing yield in WM-800 by an average of 4.47 dt/ha. A number of QTLs could be associated with the effects of known major genes in wheat, for example, the semi-dwarf genes *Rht-B1* and *Rht-D1.* In addition, a novel QTL on chromosome 2B exhibited pleiotropic effects acting simultaneously on three-grain yield components (ears per square meter, grains per ear, and thousand grain weight) and plant height. The reported QTL effects on chromosome 2B may be explained by a member of the *NITRATE TRANSPORTER 1 (NRT1)/PEPTIDE FAMILY, TaNPF5.34,* located approximately 1.05 Mb apart from the QTL. There is a need to clarify whether this genomic region is indeed based on one gene acting pleiotropically on multiple traits or if the QTL effects refer to independent genes, which are genetically linked. The ultimate answer can only be given after the hidden gene has been cloned and a transformation event or knock-out of the gene proves that the observed effects are controlled by the same gene. Further, the haplotype-based GWAS proved to be useful to identify and characterize individual founder allele effects in the multi-parental population WM-800. The WM-800 lines and QTL alleles, associated with yield and yield components, are currently implemented in breeding programs to select future nitrogen-use-efficient wheat cultivars.

## Figures and Tables

**Table 1:** Descriptive statistics of traits studied in population WM-800 and in founders.

Trait ^a^	Pop	Treatment ^b^	LSMEANS	Std Dev	Minimum	Maximum	N	CV (in %)
HEA	WM-800	Across	146.2	1.7	141.8	151.5	800	1.2
		N0	145.9	1.7	141.5	151.5	800	1.2
		N1 ***	146.5	1.8	142.0	151.5	800	1.2
	Founder	Across	146.7	1.5	143.7	148.1	8	1.0
		N0	146.4	1.5	143.4	148.0	8	1.0
		N1 ***	147.0	1.5	144.0	148.5	8	1.0
MAT	WM-800	Across	178.9	1.6	174.3	185.0	800	0.9
		N0	177.2	1.7	173.0	184.0	800	0.9
		N1 ***	180.7	2.0	175.5	186.5	800	1.1
	Founder	Across	179.5	2.0	176.2	183.0	8	1.1
		N0	177.8	1.8	174.5	180.9	8	1.0
		N1 ***	181.3	2.4	177.9	185.4	8	1.3
HEI	WM-800	Across	79.6	8.7	53.1	109.4	800	11.0
		N0	77.1	8.4	51.6	108.0	800	10.9
		N1 ***	82.0	9.2	53.9	110.7	800	11.3
	Founder	Across	79.1	4.5	74.7	89.2	8	5.7
		N0	77.2	4.5	73.1	87.5	8	5.9
		N1 ***	81.0	4.4	76.4	90.9	8	5.5
EAR	WM-800	Across	409.0	44.2	279.8	561.3	800	10.8
		N0	375.2	47.1	242.7	575.1	800	12.6
		N1 ***	442.9	59.1	297.3	649.8	800	13.3
	Founder	Across	387.5	26.0	342.2	433.3	8	6.7
		N0	354.4	24.8	320.2	384.7	8	7.0
		N1 ***	420.6	33.5	364.1	481.9	8	8.0
GNE	WM-800	Across	50.4	5.1	36.2	68.3	800	10.2
		N0	49.3	5.1	32.9	67.1	800	10.4
		N1 ***	51.5	5.7	34.6	71.2	800	11.1
	Founder	Across	51.2	3.7	46.1	56.2	8	7.2
		N0	50.5	4.1	44.8	55.6	8	8.0
		N1 *	52.0	3.5	46.5	56.9	8	6.7
TGW	WM-800	Across	42.1	3.7	31.2	53.4	800	8.8
		N0	42.7	3.5	32.8	52.6	800	8.2
		N1 ***	41.5	4.0	29.6	54.1	800	9.7
	Founder	Across	44.4	2.5	40.5	48.0	8	5.7
		N0	44.9	2.2	41.1	47.9	8	5.0
		N1 ***	43.9	2.9	39.9	48.0	8	6.6
YLD	WM-800	Across	75.5	5.3	56.9	89.3	800	7.0
		N0	70.0	5.3	48.6	84.2	800	7.6
		N1 ***	81.1	5.9	61.4	96.4	800	7.3
	Founder	Across	80.0	3.2	75.7	84.7	8	4.0
		N0	74.1	4.2	69.0	80.9	8	5.7
		N1 ***	85.9	2.3	82.4	88.9	8	2.7

^a^ Trait abbreviations: HEA, heading (in days); MAT, maturity (in days); HEI, plant height (in cm); EAR, ears per square meter (count); GNE, grain number per ear (count); TGW, thousand-grain weight (in g); YLD, grain yield (in dt/ha). Details on trait measurements are given in Table 5. ^b^ Treatments: the parameters least squares means (LSMEANS), standard deviation (Std Dev), minimum, maximum, number of observations (N), and coefficient of variation (CV) are calculated across N treatments (Across) or restricted to the reduced (N0) or standard (N1) N treatments. Significant differences between treatments are indicated by asterisks with *: *p* < 0.05 and ***: *p* < 0.001 (see ANOVA results in Table S6).

**Table 2:** Pearson correlations between seven traits studied in WM-800. (**A**) Correlation coefficients (r) based on LSMEANS calculated across both N treatments. (**B**) Correlation coefficients (r) based on LSMEANS calculated under N0 (lower triangle) and N1 (upper triangle) treatments. Trait abbreviations are given in the diagonal boxes and explained in Table 5. Significant correlations are indicated by asterisks with *: *p* < 0.05, **: *p* < 0.01 and ***: *p* < 0.001. Colors correspond to the strength of the correlation on a scale from −1 (blue) to +1 (red).

**(A)**														
	**HEA**													
**Across_N ↓**	**0.65**	***	**MAT**											
	**−0.22**	***	**−0.09**	*	**HEI**									
	**−0.00**		**0.02**		**−0.25**	***	**EAR**							
	**0.19**	***	**0.13**	***	**−0.16**	***	**−0.35**	***	**GNE**					
	**−0.21**	***	**−0.01**		**0.52**	***	**−0.36**	***	**−0.39**	***	**TGW**			
	**−0.02**		**0.13**	***	**0.14**	***	**0.02**		**0.15**	***	**0.28**	***	**YLD**	
**(B)**	**N1→**													
	**HEA**		**0.59**	***	**−0.21**	***	**0.01**		**0.18**	***	**−0.21**	***	**−0.01**	
**N0 ↓**	**0.57**	***	**MAT**		**−0.08**	*	**0.10**	**	**0.13**	***	**−0.02**		**0.20**	***
	**−0.22**	***	**−0.06**		**HEI**		**−0.26**	***	**−0.20**	***	**0.55**	***	**0.15**	***
	**−0.00**		**−0.04**		**−0.11**	**	**EAR**		**−0.26**	***	**−0.34**	***	**0.03**	
	**0.19**	***	**0.17**	***	**−0.10**	**	**−0.28**	***	**GNE**		**−0.40**	***	**0.12**	**
	**−0.20**	***	**0.04**		**0.45**	***	**−0.24**	***	**−0.33**	***	**TGW**		**0.31**	***
	**−0.03**		**0.05**		**0.13**	***	**0.10**	**	**0.17**	***	**0.24**	***	**YLD**	

**Table 3:** The number of significant marker-trait-associations (MTA) with BON_*p* < 0.05 and QTL per trait, given across N treatments and per treatment (N0 and N1).

Trait ^a^	No. of MTAs across N	R^2 b^ (%)	No. of MTAs under N0	R^2^ (%)	No. of MTAs under N1	R^2^ (%)	Total No. of QTL	No. of QTL across N ^c^	under N0	under N1
**HEA**	9	41.5	6	39.2	12	43.8	9	6 (2)	4 (1)	7 (4)
**MAT**	5	35.7	10	31.8	9	30.3	10	3 (0)	6 (5)	5 (4)
**HEI**	12	71.4	17	67.9	14	70.5	10	7 (0)	9 (2)	8 (1)
**EAR**	4	23.1	4	13.2	4	23.2	2	2 (0)	2 (0)	2 (0)
**GNE**	15	41.5	13	35.3	21	40.6	15	8 (1)	7 (3)	12 (7)
**TGW**	21	52.1	12	43.0	18	53.4	13	12 (2)	7 (1)	10 (4)
**YLD**	14	34.9	9	30.2	11	34.8	12	8 (2)	5 (2)	7 (5)
**∑**	80		71		89		71	46 (7)	40 (14)	51 (25)
	240							137 (46)		

^a^ Trait abbreviations: heading (HEA), maturity (MAT), plant height (HEI), ears per square meter (EAR), grain number per ear (GNE), thousand grain weight (TGW), and grain yield (YLD). ^b^ The explained genotypic variance (R^2^) was calculated using all significant HT. ^c^ QTL were assigned to one of the three categories (N0, N1, and across N treatments). Numbers in brackets indicate QTL solely detected across N, under N0 and N1 treatments, respectively.

**Table 4:** Conditions of WM-800 field trials carried out in seven environments.

Abbreviation of Environment ^a^ (Location_year)	Plot Size (m^2^)	Soil Score ^b^	Soil Nmin ^c^ (kg ha^−1^)	Applied N ^d^ (kg ha^−1^)		Time Point of N Application ^e^		N Difference between N0 & N1 (kg ha^−1^)
				N0	N1	N0	N1	
**HAD_17**	5.70	92	85	60	100	1	1–2	40
**HAD_18**	5.70	95	124	0	140	none	1; 2; 4	140
**HAL_17**	5.70	65	45	15	155	1	1–2	140
**HAL_18**	5.70	38	60	40	165	1	1–3	125
**MOS_18**	7.20	75	28	80	210	1–2	1–3	130
**SEL_17**	6.45	80	88	60	205	1	1–4	145
**SEL_18**	6.45	85	57	50	190	1	1–4	140

^a^ Locations: HAD, Hadmersleben; HAL, Halle; MOS, Moosburg; SEL, Seligenstadt; Years: 17, 2016/17; 18, 2017/18. ^b^ Official German score for the estimated fertility and productivity of the cultivated soil on a range from 0 to 100 (www.destatis.de, accessed 18 October 2022). ^c^ Mineralized soil N (=Nmin) was measured by extracting ammonium and nitrate N with calcium chloride from a 30 cm soil sample taken in February of each trial year. ^d^ Total amount of nitrogen fertilizer applied under each treatment. Ammonium sulphate nitrate (time point 1) and calcium ammonium nitrate (time points 2–4) were applied as N fertilizers. ^e^ The N fertilizer application took place at the following time points: 1: beginning of shooting (BBCH 30–31, [105] in March, 2: end of shooting (BBCH 37–39) [105] in April, 3: flowering (BBCH 61–65) in May and 4: beginning of grain filling (BBCH 71) in June.

**Table 5:** List of seven evaluated traits.

Abbr.	Trait	Unit	Method of measurement	No. of E ^a^
**HEA**	Heading	days	Number of days since the 1st of January when half of the ear emerged (BBCH 55, [105] for 50% of plants in a plot	6
**MAT**	Maturity	days	Number of days since the 1st of January until grains reached hard dough stage where grain content is firm and fingernail impression is held (BBCH 87, [105] for 50% of plants in a plot	2
**HEI**	Plant height	cm	Recorded as the distance from the ground to the tip of the erected ear	7
**EAR**	Ears per square meter	Count	Measured from half a meter of one row per plot and converted into the number of ears per square meter	3
**GNE**	Grain number per ear	Count	Recorded from 10 harvested ears per plot with MARVIN seed analyzer (GTA Sensorik GmbH, Germany)	4
**TGW**	Thousand grain weight	g	Calculated after harvest with MARVIN seed analyzer (GTA Sensorik GmbH, Neubrandenburg, Germany) based on a minimum of 300 seeds per plot	7
**YLD**	Grain yield	dt/ha	Grain harvest of field plot converted into dt/ha	7

^a^ Number of environments tested.

## Data Availability

Data supporting the findings of this study are available within the paper and the supplementary materials published online. Additional supporting data, concerning the development of haploblocks and haplotypes in WM-800, are available at DRYAD (https://doi.org/10.5061/dryad.zcrjdfnfk).

## Supplementary Materials

The following supporting information can be downloaded at: https://www.mdpi.com/article/10.3390/plants11243508/s1, Figure S1: Histograms of phenotypic trait distribution in WM-800 across and under N0 and N1 treatments; Figure S2: Circular Manhattan plots of genome-wide association scans for seven traits studied in WM-800 under N0 and N1 treatments, respectively; Table S1: List of founder varieties of the eight-way MAGIC-WHEAT population WM-800; Table S2: Weather conditions at test locations; Table S3: Trait raw data of WM-800 lines and founders collected in seven environments under two N treatments (N0 and N1); Table S4: Trait LSMEANS of WM-800 lines and founders estimated across N treatments and per N treatment; Table S5: Pivot table based on Table S4, Trait LSMEANS of WM-800 lines and founders estimated across N treatments and per N treatment; Table S6: ANOVA results for 7 traits studied, separately for MAGIC population (N = 800) and founders (N = 8); Table S7: Decomposition of variances and estimation of heritabilities for 7 traits, across and within treatments; Table S8: List of 240 significant marker-trait associations (MTAs) and effect estimates with BON_*p* < 0.05 for seven traits studied across N treatments and per N treatment; Table S9: Pivot table based on Table S5. List of 240 significant marker-trait associations (MTAs) and effect estimates with BON_*p* < 0.05 for seven traits studied across N treatments and per N treatment; Table S10: List of 71 QTL and effect estimates at 137 significant haploblocks or singular SNPs with BON_*p* < 0.05, regulating seven traits studied across N treatments and per N treatment; Table S11: Selection of informative parameters (out of 12 yield QTL and 6 quantitative traits) in order to predict YLD, YLD_N0 and YLD_N1 by stepwise regression; Table S12: Prediction of YLD, YLD_N0, and YLD_N1 based on stepwise regression models, selected in Table S11.
